# Complexities of road safety interventions in Low and Middle Income Countries (LMICs) posed a major challenge in achieving decade goal-lessons for the next span 

**Published:** 2019-08

**Authors:** AKM Fazlur Rahman, Farah Naz Rahman

**Affiliations:** ^*a*^Centre for Injury Prevention and Research, Bangladesh.

## Abstract

**Background:**

Most of the Low and Middle Income Countries in the world unsucessful to reduce the fifty percent of the Road Traffic Injury (RTI) deaths, which was target set in the last decade goals (2011-2020). This failure is largely due to the result of complexities of the problem itself, its causes, designing and implementing interventions and evaluation of Road Traffic Injury in low resource settings. The paper aimed to explore the nature of these complexities and provide a direction to address the RTIs efficiently in next decade in LMICs.

**Methods:**

Review of literatures related to the RTI prevention in LMICs especially nature of problem, factors related to RTIs and policy & interventions. Special emphasis was given on the complexity of road transport system, perception of road safety issues, road user’s behavior, public health system and policy maker’s behavior in LMIC context. The Global Status Reports on Road Safety were critically analyzed to explore the complexities of road safety related to low resources.

**Results:**

The complexities related to the RTI prevention in LMICs are mostly expressed on its i) incorrect perception of the problem, ii) factors related to RTIs, iii) multi-dimensional preventive approach, iv) inequalities in health and well-being, v) complicated evaluation modalities. Factors linked to incorrect perception of RTI problem are insufficient information, societal insights about the RTIs and health system’s inability to capture the RTI events. Complex risk factors of RTIs related to demographic, social, environmental, and economic and inequity that exists in low resource settings and most of them are interlinked. There is no one- single approach effective for RTI prevention.

**Conclusions:**

Although RTI is a leading killer of productive peoples in many LMICs, it failed to create enough importance in national and global policy issues as magnitude and causes and interventions of RTIs are unclear, and complex. So the complex RTI landscaping hinders in priority setting, resource allocation and prevention efforts. It is high time to reinvent new strategies beside existing preventive approach for RTIs prevention considering all these complexities in LMICs through intensive research.

**Keywords:**

Road Traffic Injuries, Low and Middle Income Countries, Complexities of problem, Management

